# Hypoglossal dural arteriovenous fistula: a rare cause of unilateral hypoglossal nerve palsy

**DOI:** 10.1259/bjrcr.20160144

**Published:** 2017-03-10

**Authors:** Nathan Howard Ho Leung Chan

**Affiliations:** Department of Anatomy and Human Sciences, King's College, University of London, London, UK

## Abstract

Ms Y, a 57-year-old female presented with a 1-week history of tongue deviation. The history of the presenting complaint also included minor dysarthria, dysphagia for solids and liquids as well as a 2- to 3-month history of pulsatile tinnitus affecting the right ear. Examination of the cranial and peripheral nerves revealed a right hypoglossal nerve lower motor neurone palsy. MRI demonstrated a dural arteriovenous fistula (DAVF) in the region of the right hypoglossal canal. She underwent a cerebral angiogram, which confirmed a hypoglossal DAVF with predominant supply from the neuromeningeal branches of the right ascending pharyngeal artery. She has been able to cope with her symptoms and remains on active surveillance. Hypoglossal nerve palsy is uncommon, causes may be classified according to location. DAVFs are a rare cause of hypoglossal nerve palsy. DAVFs can be graded according to their pattern of venous drainage. This case illustrates the complex venous anatomy of the craniocervical junction, which enables postural-dependent drainage through the internal jugular and vertebral venous systems. This network of veins is encountered during interventional radiology procedures and neurosurgical skull base approaches.

## Clinical presentation

Ms Y, a 57-year-old female presented with a 1-week history of tongue deviation. She first presented to primary care in October 2015 and was referred directly to Accident and Emergency. Her medical history includes a traumatic pneumothorax, treated with video-assisted thoracoscopic pleurodesis and bullectomy in May 2015. The history of the presenting complaint also included minor dysarthria, mild dysphagia for solids and liquids, as well as a 2– to 3-month history of pulsatile tinnitus affecting the right ear.

**Figure 1.**
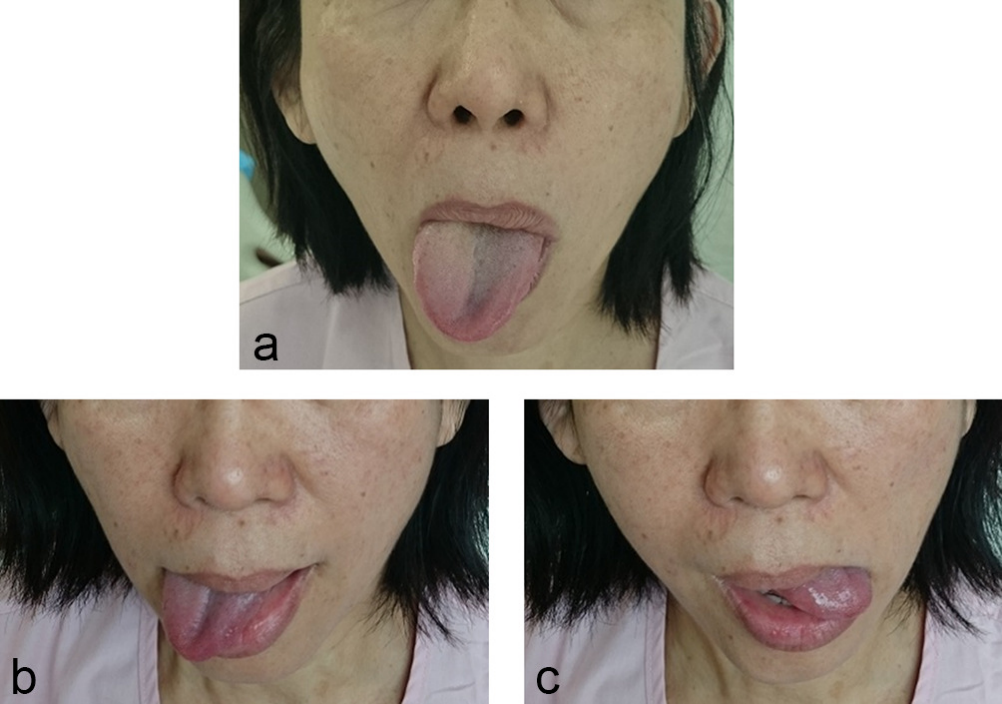
Ms Y’s tongue position when instructed to protrude her tongue (a), images below illustrate extent of lateral movement to the right (b) and left (c).

Examination of the cranial and peripheral nerves revealed a right hypoglossal lower motor neurone palsy. The tongue deviated to the right when protruded and there was associated atrophy and weakness affecting the right half of the tongue (Figure 1). Taste sensation, speech, swallowing and gag reflex were all intact. Bilateral trapezius and sternocleidomastoid muscle power and bulk were normal. There were no other cranial nerve deficits and systemic examination was unremarkable.

## Differential diagnosis

Causes of hypoglossal nerve palsy may be divided according to the location of the lesion in relation to the course of the hypoglossal nerve.^1^ The hypoglossal nerve arises from its nucleus in the medulla as a series of rootlets emerging in the preolivary sulcus. The rootlets lie posterolateral to the vertebral arteries in the premedullary cistern, they enter the hypoglossal canal and merge to form the hypoglossal nerve, which continues extracranially in the nasopharyngeal carotid space, deep to the internal jugular vein, internal carotid artery, glossopharyngeal and vagus nerves. The nerve passes inferiorly and laterally, to lie between the internal carotid artery and jugular vein, superficial to the vagus nerve. At the angle of the mandible, the nerve loops anteriorly lying inferior to the posterior belly of the digastric muscle. At the level of the hyoid bone, the nerve crosses the lingual artery and curves forwards to enter the sublingual space, between the mylohyoid and hyoglossus muscles, before penetrating the genioglossus muscle.

Common causes of hypoglossal nerve palsy divided according to location^1^

1. Medullary segment: The nucleus of the hypoglossal nerve may be affected by a number of pathologies within the medulla. Common pathologies include infarction, haemorrhage, demyelination, e.g. multiple sclerosis, as well as tumours of the brainstem, both primary, e.g. brainstem glioma and secondary metastatic deposits. Disease in this region may involve other lower cranial nerves and it is important that these are fully assessed at presentation. Disease crossing the midline may lead to complete paralysis of the tongue, resulting in airway obstruction.
2. Cisternal segment: The rootlets of the hypoglossal nerve may be susceptible to several pathological processes in the premedullary cistern. Given the close proximity of the vertebral arteries, aneurysms or dolichoectasia of these vessels may result in direct compression. Intracranial extension of skull base neoplasms, such as chordomas or meningiomas may affect the nerve. Meningitis involving the brainstem, especially tuberculous meningitis, or an organising subarachnoid haemorrhage, can lead to nerve dysfunction. Rheumatoid arthritis affecting the odontoid process may cause nerve impingement or stretching of the rootlets.
3. Skull base segment: The hypoglossal nerve may be compromised within the hypoglossal canal by tumours and trauma. Typical primary tumours affecting the skull base include chordomas, chrondrosarcomas, plasmacytomas and meningiomas of the skull base. Metastases are most commonly from lung, breast, or prostate. Direct extension of nasopharyngeal carcinoma or cholesteatoma may also occur. Basal skull fractures may extend into the occipital condyle and hypoglossal canal. Rarely, dural arteriovenous fistulas (as in this case) may cause mass effect on the nerve within the hypoglossal canal.
4. Carotid space segment: Injury within the carotid space is most commonly due to malignancy, but may be secondary to a variety of other insults. Primary and nodal squamous cell carcinoma, salivary gland malignancies, lymphoma, sarcomas, and extra-nodal metastatic disease may all affect this area. Benign lesions e.g. lipomas and paragangliomas; vascular diseases *e.g.* carotid artery dissection, jugular thrombosis; and iatrogenic causes e.g. radiotherapy, complicated carotid artery dissection, can also result in nerve dysfunction. Infectious processes, originating from surrounding structures, may spread via fascial planes to involve this space. Penetrating trauma, in the form of stab or gunshot wounds, can affect the nerve in this relatively superficial region.
5. Sublingual segment: Carcinomas, in particular invasive squamous cell carcinoma, are the most common cause of hypoglossal nerve palsy in this segment and usually arise from the base or oral portion of the tongue. Salivary gland tumours occur less commonly, but may also affect the distal portions of the nerve.

## Investigations/Imaging findings

An initial unenhanced CT scan was normal, with no evidence of acute intracranial haemorrhage or infarction. Further history taking and examination elucidated subtle symptoms suspicious for a vascular lesion, in particular, pulsatile tinnitus, most noticeable at night, which is a common symptom of dural arteriovenous fistulas.^2^ This occurred over a 2- to 3-month period, preceding the 1-week history of hypoglossal nerve palsy.

**Figure 2.**
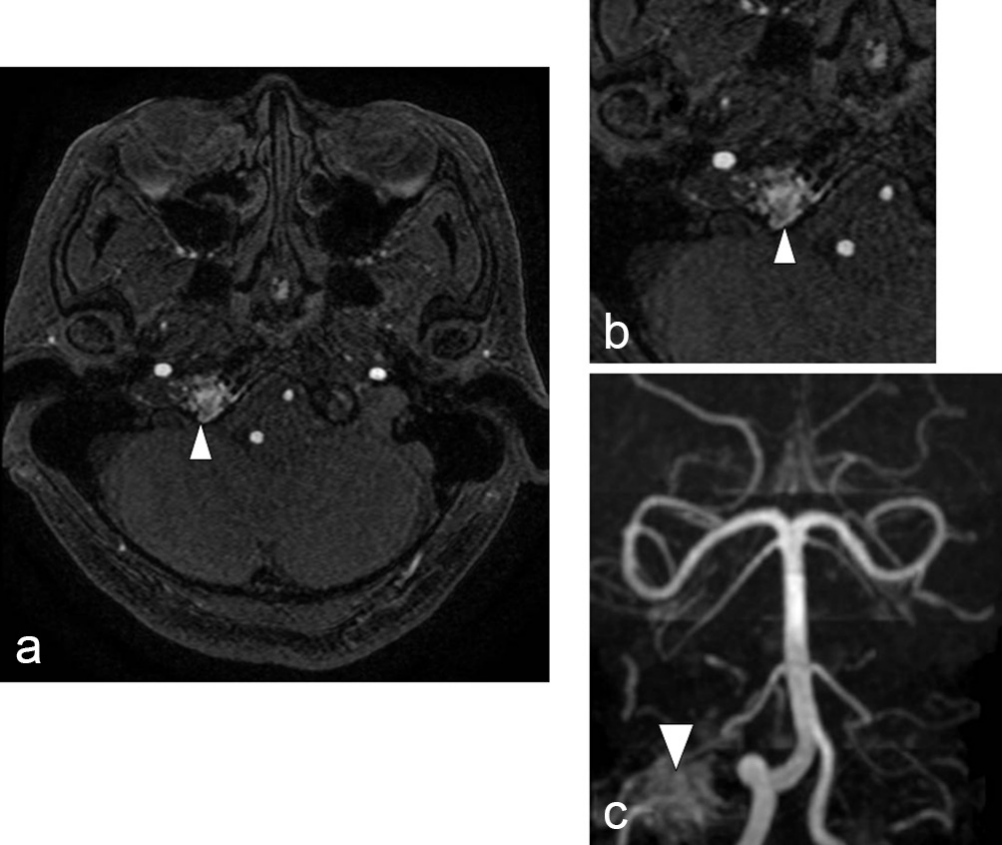
Axial MRA, time of flight sequence (a) magnified view (b) a region of high signal intensity is visible in the region of the right hypoglossal canal (white triangle – HCDAVF). AP 3D reconstruction of MRA sequences (c) illustrates the relation of the DAVF to the right posterior inferior cerebellar artery, which appeared, in this sequence, to be supplying the DAVF, clarification following catheter angiogram revealed this was not the case (see Figures 3 and 4).

Following discussion with the neuroradiology team, an MRI with MRA, MRV and contrast sequences was arranged, to assess regional neurovascular structures. This demonstrated an area of high signal on the time of flight MRA sequence, in the region of the right hypoglossal canal, which corresponded to areas of low signal, in keeping with flow void, on both *T*_1_ and *T*_2_ weighted sequences. The right hypoglossal canal was widened, with intermediate signal on *T*_1_, low signal on *T*_2_ and enhanced following contrast administration, suggesting a prominent venous structure. MRV revealed mild dilatation of the right jugular bulb, near its junction with the right sigmoid sinus, but no connection with the venous structure. There was no evidence of intracranial mass or venous thrombosis. Collectively, the imaging findings suggested a DAVF in the region of the right hypoglossal canal (Figure 2).

**Figure 3.**
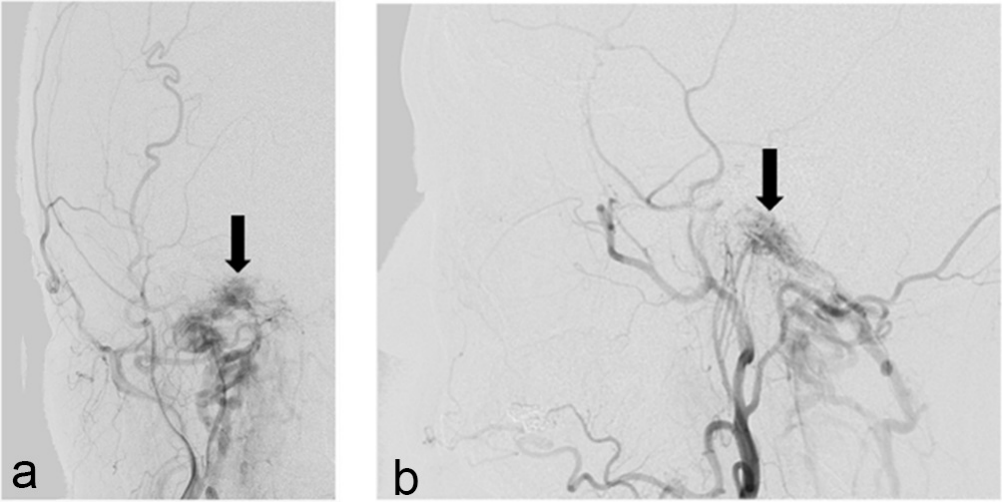
Right external carotid artery injection AP (a) and lateral (b), demonstrating HCDAVF (black arrow), with arterial supply mostly from the ascending pharyngeal artery. Anterograde venous drainage is seen directed inferiorly into the internal and external vertebral venous plexuses and deep cervical veins (see Figure 4 and text for more details).

Ms Y was transferred to a tertiary centre and underwent an angiogram of the bilateral internal, external carotid and vertebral arteries (Figure 3). This confirmed a skull base AVF with predominant supply from the right external carotid artery branches, including the ascending pharyngeal artery, as well as contributions from the right internal carotid and vertebral arteries. Minimal crossover supply was noted from the left external and internal carotid arteries, with no contributions from the left vertebral artery. Venous drainage was directed inferiorly into the internal and external vertebral venous plexuses (VVPs), with no retrograde flow into venous sinuses, cerebellar or perimedullary veins. No connection was found between the DAVF and the ipsilateral jugular venous system. Overall, the study confirmed the presence of a right skull base AVF, with predominant supply from the right ascending pharyngeal artery.

## Outcome and follow up

The management options, including embolization, surgery and active surveillance with serial imaging, were discussed. Ms Y was reviewed by both a neuro-occupational therapist and physiotherapist, who provided her with tongue exercises.

**Figure 4.**
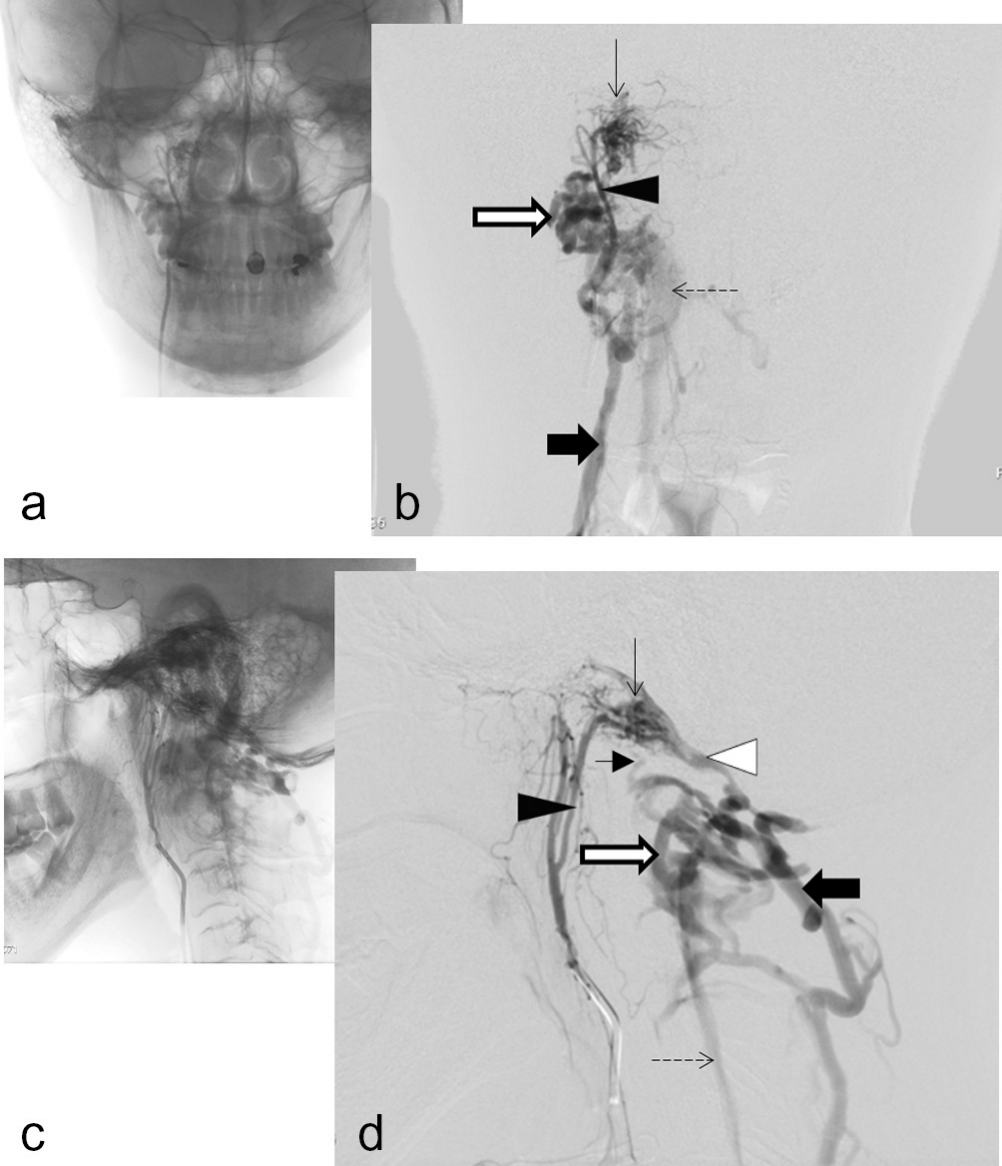
Right ascending pharyngeal artery digital subtraction angiogram AP (b), with pre-subtraction image (a) and lateral views (d) with pre-subtraction image (c) demonstrate a HCDAVF (thin black arrow), arising from the meningeal branch of the ascending pharyngeal artery (black triangle), draining via the venous plexus of hypoglossal canal (white triangle) and lateral condylar veins (short black arrow) into the vertebral artery venous plexus (white arrow), the anterior internal vertebral venous plexus (dashed arrow) and the deep cervical veins (black arrow).

Ms Y opted for active surveillance and a repeat angiogram was performed a month later in November 2015 (Figure 4). The DAVF remained stable in size and was further characterized as arising from the neuromeningeal branches of the right ascending pharyngeal artery, at the level of the anterior condylar confluence, with venous drainage directed inferiorly, via the condylar veins, into the VVPs and deep cervical veins.

Her symptoms have remained unchanged and she still suffers from a right hypoglossal nerve palsy, as well as pulsatile tinnitus. However, she has been able to cope with these symptoms and lives a normal life. In view of this, she has been treated conservatively and remains on 6-month follow up.

## Discussion

Dural arteriovenous fistulas (DAVFs) are shunts connecting dural arteries to dural venous sinuses, meningeal veins or cortical veins. They account for 10–15% of intracranial arteriovenous malformations.^2^ They are commonly described according to the venous sinus to which they are related or the anatomic location of the fistula.

DAVFs are graded according to their pattern of venous drainage, examples of classification schemes include the Borden classification and Cognard classification. In general, lesions that drain into cortical veins and those associated with retrograde flow are associated with a higher risk of haemorrhage and non-haemorrhagic neurological deficit.^2^ This DAVF did not exhibit retrograde flow or cortical venous drainge and represents a Borden Type 1, Cognard Type 1 lesion.

Hypoglossal canal DAVFs (HCDAVFs) account for 3.6–4.2% of DAVFs and involve the anterior condylar confluence (ACC) and/or venous plexus of hypoglossal canal (VPHC) (Terminologia Anatomica—international anatomical nomenclature), also known as the anterior condylar vein (ACV). They may occasionally present with hypoglossal nerve palsy (11.7% of HCDAVFs), which has been attributed to pulsatile compression and venous hypertension from a dilated VPHC within the hypoglossal canal.^3^

HCDAVFs are classified into three categories according to their pattern of venous drainage, which correspond with distinct clinical presentations: Type 1—dominant anterograde venous drainage to IJV and/or VVP with or without reflux to transverse, sigmoid, inferior petrosal or cavernous sinus; Type 2—dominant retrograde drainage to the cavernous sinus and/or orbital veins with or without anterograde drainage to the IJV and/or VVP or cortical venous reflux; and Type 3—dominant or exclusive venous drainage to cerebellar pial or perimedullary veins.^3^

The case described above, belongs to the Type 1 category, which usually presents with symptoms of pulsatile tinnitus, owing to temporal bone conduction of the venous bruit to the inner ear. These may occasionally present with hypoglossal nerve palsy or involuntary tongue movements. Type 2 HCDAVFs present with orbital symptoms e.g. chemosis, proptosis and/or diplopia from oculomotor or abducens nerve palsies, owing to reflux into the cavernous sinus and superor orbital vein. Type 3 HCDAVFs are more likely to develop myelopathy and intracerebral haemorrhage.^3^

Treatment options of HCDAVFs include endovascular therapy, surgery or radiotherapy, as well as combinations of all three. At present there is insufficient data to advocate one treatment over the other, especially since the number of surgical and radiotherapy cases are few. Endovascular therapy may be trans-arterial or trans-venous, using coils, liquid embolic agents or a combination of both. Trans-venous coil embolization via the internal jugular vein (IJV) is generally preferred and has a high rate of clinical cure, 61 of 67 cases in a recent review by Spittau et al (2014). However this risks hypoglossal nerve palsy as a complication (3 of 68 cases), likely due to mass effect of the coils on the hypoglossal nerve.^3,4^

Trans-arterial approaches and trans-venous methods involving liquid embolic agents such as polyvinyl alcohol (PVA), onyx or N-butyl cyanoacrylate (NBCA) risk embolization of the ascending pharyngeal artery, leading to lower cranial nerve palsies (IX–XII), due to supply of the vasa-nervorum by the jugular branch.^3,5^ In general, surgery involving ligation of arterial feeders or transection at the dural exit of the vein is associated with a higher rate of morbidity and mortality (> 10%),^2^ and should be reserved for cases where endovascular therapy is not feasible.

**Figure 5.**
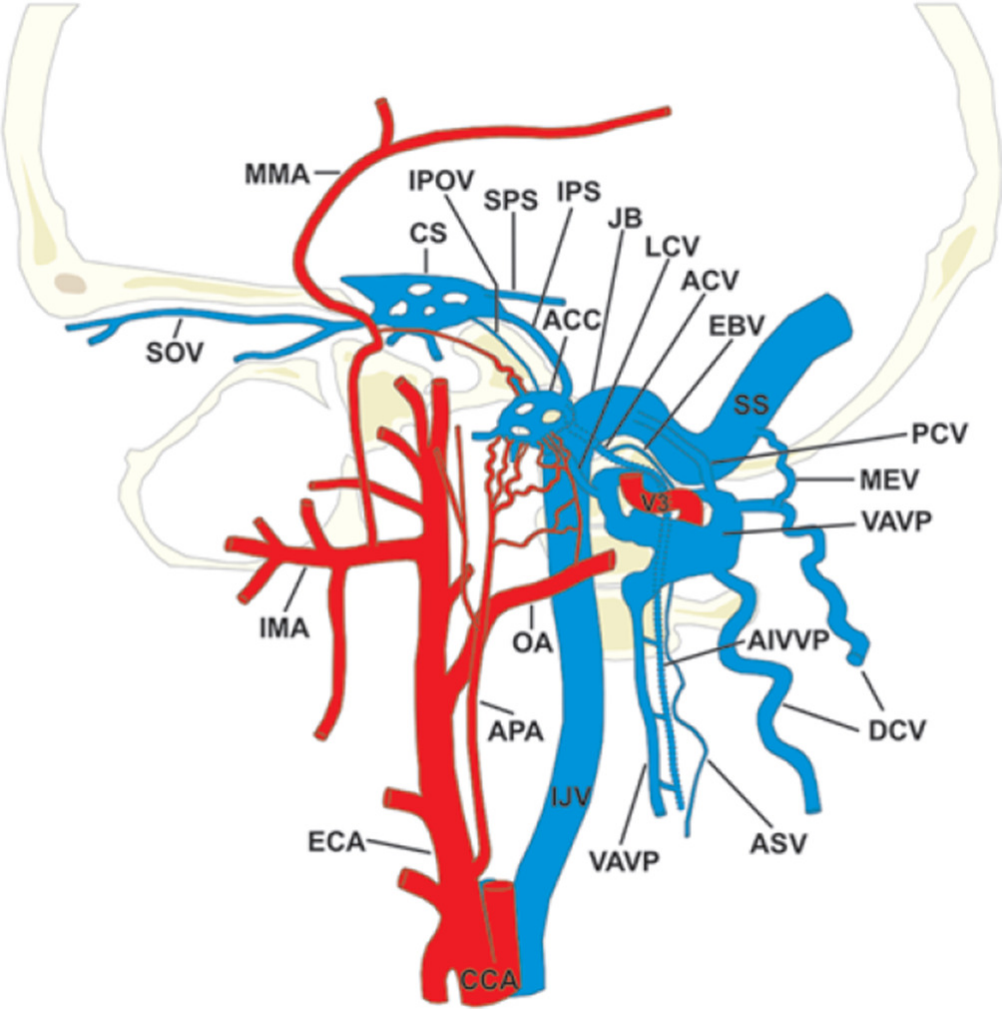
Illustration of HCDAVFs with the fistula points at the ACC (lateral view). Arterial feeders are depicted from the neuromeningeal division of the ascending pharyngeal artery (APA), petrous branch of the middle meningeal artery (MMA), and the mastoid branch of the occipital artery (OA). Variable venous connections of ACC and condylar veins with possible venous drainage routes are also depicted. ACV = VPHC venous plexus of hypoglossal canal / anterior condylar vein; AIVVP = anterior internal vertebral venous plexus; APA = ascending pharyngeal artery; ASV = anterior spinal vein; CCA = common carotid artery; CS = cavernous sinus; DCV = deep cervical vein; EBV = emissary bridging vein; ECA = external carotid artery; JB = jugular bulb; IMA = internal maxillary artery; IPOV = inferior petro-occipital vein; IPS = inferior petrosal sinus; LCV = lateral condylar vein; MEV = mastoid emissary vein; MMA = middle meningeal artery; OA = occipital artery; PCV = posterior condylar vein; SOV = superior ocular vein; SPS = superior petrosal sinus; SS = sigmoid sinus; VAVP = vertebral artery venous plexus. Copyright Journal of Neurosurgery, published with permission.

This rare case illustrates the complex venous anatomy at the cranio-cervical junction (Figure 5). Venous drainage of the intra-cranial compartment occurs via numerous collateral pathways, maintaining homeostasis in a variety of physiological conditions. The internal jugular system is the dominant outflow in the supine position, which contrasts with the erect position where there is little flow in the IJVs and the majority of flow occurs via the VVPs.^6–8^ In a study, where measurements of venous blood flow in the neck, were taken with the body at various degrees of incline, vertebral venous outflow was found to surpass IJV outflow at 45 degrees.^7^ A separate study found that in 28% of individuals, between zero and two thirds of total arterial flow in the supine position, was returned by the IJVs, suggesting significant contribution from alternative venous drainage pathways, even in the supine position.^6^

In recent years the anatomy of the venous network at the cranio-cervical junction has been studied by different groups,^9–12^ resulting in variation in anatomical nomenclature and definitions. A cadaveric corrosion cast study, combined with MR venography, performed by Ruiz et al (2002), identified the numerous connections of the anterior condylar confluence (ACC), an important venous bridge between the dural venous sinuses of the posterior cranial fossa, internal jugular veins and vertebral venous systems. This is also the point of fistulation, in this case of HCDAVF (Figure 4).

The ACC communicates with the cavernous sinus via its connections with the inferior petrosal sinus and the venous plexus of the internal carotid artery (Figure 5). It is continuous with the basilar venous plexus and marginal sinus by way of the VHPC (labelled as ACV in Figure 5), which drains inferiorly into the anterior internal vertebral venous plexus (AIVVP).^3^ It is connected to the IJVs by the lateral condylar vein (LCV) and by one or several branches from the IJV or bulb, these small connections are not shown in Figure 5.

In conclusion, HCDAVFs may be classified into 3 categories according to their pattern of venous drainage, which correspond with distinct clinical presentations. Treatment may involve endovascular interventions, with a preference for trans-venous methods, surgery and radiotherapy, in combination or alone. This case illustrates the complex venous anatomy of this region, which has important implications in skull base surgery and interventional neuroradiology.

## Learning points

1. Causes of hypoglossal nerve palsy may be divided according to location in relation to the course of the hypoglossal nerve.
2. Hypoglossal dural arteriovenous fistula is a rare cause of unilateral hypoglossal nerve palsy, which involves the anterior condylar confluence or venous plexus of hypoglossal canal.
3. DAVFs are classified according to their pattern of venous drainage.
4. DAVFs can be treated by endovascular therapy, surgery or radiotherapy, as well as combinations of all three. Endovascular therapy is the preferred option and may be trans-arterial or trans-venous, using coils, liquid embolic agents, or a combination of both. In this case trans-venous coil embolization is the first line treatment.
5. The veins of the craniocervical junction form a complex intricate network, the anatomical knowledge of which is important in neurosurgical and interventional radiology procedures.
6. Drainage of the intracranial compartment occurs via both the internal jugular veins and the vertebral venous plexus in manner that exhibits postural dependence.
7. The anterior condylar confluence is a venous crossroad located at the extracranial aperture of the hypoglossal canal, connecting the dural venous sinuses, internal jugular and vertebral venous systems.

## Consent

Written informed consent was obtained from the patient for publication of this case report, including accompanying images.
